# Levels and correlates of pandemic anxiety in people living with rare diseases: a cross-sectional analysis using a structural equation model

**DOI:** 10.1186/s13023-025-04152-x

**Published:** 2026-01-27

**Authors:** Hermann Siebel, David Zybarth, Laura Inhestern

**Affiliations:** 1 Department of Education and Psychology, Freie Universität Berlin, Habelschwerdter Allee 45, 14195 Berlin, Germany; 2 Department of Medical Psychology, University Medical Center Hamburg-Eppendorf, Martinistraße 52, 20246 Hamburg, Germany; 3 Partner Site Hamburg, German Center for Child and Adolescent Health, Martinistraße 52, 20246 Hamburg, Germany

**Keywords:** Pandemic anxiety, Rare diseases, Structural equation modeling

## Abstract

**Background:**

Within the context of the COVID-19 pandemic and beyond, pandemic anxiety (PA) is of high social and psychopathological relevance. Compared to the general population and people living with common diseases, people living with rare diseases suffer more from the effects of a pandemic in various areas. To date, there are almost no systematic studies on PA in this subpopulation. Therefore, the current study examines the levels and factors associated with PA, as well as the relationship of PA with clinical measures of anxiety and depression disorders in people living with a wide range of different rare diseases.

**Methods:**

Data are drawn from an online survey conducted between March 2022 and February 2023 at the Department of Medical Psychology, University Medical Center Hamburg-Eppendorf, on the care situation of people living with rare diseases during the COVID-19 pandemic. Analyses include descriptive statistics, Welch’s t-tests, linear regressions, and a multivariate mediation model tested via structural equation modeling ($N = 590$).

**Results:**

Compared to the general population, people living with rare diseases are more affected by PA during a pandemic. Female gender, age of 50 years and older, and living alone are risk factors for particularly high levels of PA. Furthermore, a previous COVID-19 infection is associated with lower PA; receiving vaccination correlates with higher PA. In addition to sociodemographic factors, health-related quality of life (HRQOL) and daily burden due to the rare disease are significantly associated with PA levels. Moreover, an increase in PA is associated with increased anxiety and depression scores in clinical screening questionnaires. Lastly, PA mediates the links of daily burden with anxiety and depression disorders.

**Conclusions:**

Findings highlight specific factors that should be addressed to effectively improve the situation of people living with rare diseases in the likely event of another pandemic. In addition, it becomes apparent that PA has negative implications for mental health that can persist beyond the context of a pandemic. Hence, PA should not be trivialized as a temporary pandemic state. More research is needed to compensate for the limitations of the present study and to better understand the structure of PA in people living with rare diseases.

## Background

In December 2019, the first confirmed cases of SARS-CoV-2 marked the beginning of a global pandemic that, by the end of 2024, led to almost 780 million infections and more than 7 million deaths worldwide [1]. Beyond physical health, the COVID-19 pandemic disrupted economic, social, and psychological well-being to a greater extent than previous pandemics. Since more than two-thirds of the global population are now vaccinated against SARS-CoV-2 and daily infection, hospitalization, and mortality rates have significantly declined compared to peak phases (2020 to 2022) [1], the COVID-19 pandemic is considered by many to be over. However, research indicates that the likelihood of similar epidemics and pandemics occurring in the near future is relatively high [2, 3], and is steadily increasing due to the climate crisis [4]. Hence, systematic investigation of pandemic-related mental health and its risk and protective factors remains highly relevant, even beyond the scope of COVID-19.

Extensive evidence highlights the significant impact of the COVID-19 pandemic on mental health, including increased rates of (post-traumatic) stress, anxiety, depression, loneliness, sleep disturbances, and suicidal ideation [5–8]. Longitudinal studies indicate that, compared to pre-pandemic levels, symptoms worsened significantly during the pandemic and are likely to persist throughout and beyond its duration [9–12]. Anxiety is one of the most affected domains. A meta-analysis shows that during the pandemic, global point prevalence rates reached 27% (95% CI: 23.7%; 32.1%) in the general population and 40% (95% CI: 30.1%; 50.1%) among those infected with COVID-19 [13]. This corresponds to a tripling of pre-pandemic rates [14] and exceeds anxiety levels reported during previous epidemics [15]. Importantly, the rise in anxiety symptoms has not been matched by a proportional increase in diagnoses of full anxiety disorder [16]. This discrepancy suggests the emergence of a context-specific phenomenon, often conceptualized as pandemic anxiety (PA).

Initially framed through clinical diagnostic categories such as (agora-) phobia, PA was soon recognized as a distinct phenomenon not adequately captured by conventional classifications [17, 18]. Although there are overlaps with common anxiety disorders, PA is multifaceted and can be more adequately described as a transdiagnostic construct [19, 20]. In response, several scales were developed to measure this construct (see [18, 21] for an incomplete overview). Among them, the Pandemic Anxiety Scale (PAS) was created as a concise alternative to large uneconomical scales, capturing two core dimensions of pandemic-related anxiety: health anxiety and anxiety about broader consequences [22]. Thus, it defines PA as anxiety-related symptoms arising in response to pandemic-specific aspects and contextual factors [22].

People living with rare diseases represent a vulnerable subgroup during pandemics. In the European Union, a disease is classified as rare if it affects no more than 50 in 100,000 people (0.05%). Although each rare disease is uncommon, approximately 6,000 distinct conditions have been identified globally, with around 250 added annually. As a result, an estimated 263 to 446 million people worldwide (4 to 6% of the global population) live with a rare disease at any given time [23]. Rare diseases are typically complex, chronic, and incurable, associated with reduced life expectancy, increased risk for mental health disorders, and significantly lower health-related quality of life (HRQOL) compared to the general population and those with common chronic illnesses [24, 25]. Access to specialized care remains limited due to a shortage of (medical) experts for rare diseases, leading to delayed diagnoses and inadequate treatment options, which are often expensive and difficult to obtain [26, 27]. These healthcare barriers are compounded by daily stressors such as stigma, financial strain, social exclusion, and lack of public awareness or understanding [28].

Medical conditions increasing the risk of severe COVID-19 cases and deaths include cardiovascular diseases, chronic lung conditions, and immune deficiencies [29]. Most rare diseases fall into these categories [30], making patients with rare diseases a medically high-risk group in the context of COVID-19. In contrast to more common (e.g., respiratory) diseases also increasing the likelihood of severe COVID progression, people with rare diseases already face the additional challenge of limited medical access and expertise (see above) regardless of a pandemic. Studies confirm increased rates of severe symptoms and mortality among patients with rare diseases [31, 32], with one population-based study reporting a 4.5-fold higher COVID-19 mortality rate in this group [33]. Beyond physical vulnerability, people living with rare diseases show impaired mental health outcomes during the pandemic, including elevated stress, anxiety, depression, loneliness, and isolation [34]. Not all rare diseases qualify as COVID-19 risk conditions, nor are all associated with a degree of disability [30]. Still, comparisons with healthy control groups reveal significantly increased anxiety and depression levels among patients with rare disease [35, 36].

These findings are not surprising, considering that the above mentioned shortcomings related to rare disease care are further exacerbated by negative pandemic impacts. In times of the COVID-19 pandemic, for example, there was an even greater difference in some dimensions of HRQOL among patients with rare diseases compared to the general population [27] than before. Pre-existing barriers to medical care worsened, with reports of therapy disruptions, limited access to medication, protective equipment, and nursing services, as well as closure of specialized clinics due to infection risks [37, 38]. In a survey of roughly 3500 patients with a rare disease, 16% were denied hospital admission in cases that would normally require it [32]. Additionally, 63% of rare disease advocacy organizations reported pandemic-related impairments, often due to funding cuts [39]. Daily stressors intensified as well, particularly financial worries [40]. Many patients with rare diseases also faced COVID-related stigma [27], for example when unable to wear masks or get vaccinated due to medical conditions, and felt ignored when others refused protective measures without valid reasons [41]. All these specific mechanisms can be presumed to mediate the disproportionately negative effect of the COVID-19 pandemic on the mental health of people living with rare diseases.

As previously pointed out, PA is a relevant dimension of mental health in times of a pandemic. So far, PA has often been measured using standard clinical anxiety scales, with pandemic effects inferred solely from timing. Particularly in the context of rare diseases, systematic research on levels and correlates of PA, using standardized instruments that conceptualize PA as a distinct construct and therefore allow for more valid assessment of these pandemic-related anxiety responses, is rare. Regarding PA levels, so far there is one study that uses a standardized PA scale to compare a small rare disease sample to the general population. Expectedly, significantly higher PA was found among 58 individuals with different rare diseases compared to Norwegian normative values used for standardization of the scale (Cohen’s d = 0.53) [42]. To replicate this finding in a larger sample, the following hypothesis was derived:

**H1**: PA is higher among the study sample than in the German general population.

Regarding correlates of PA, a few studies measure PA using standardized scales, most of them focusing on general population samples. Women consistently report higher PA than men across various studies and scales [22, 43–46], with similar findings in rare disease samples [47, 48]. Regarding age, some studies report higher PA among older adults [49, 50], which could also be replicated in a rare disease sample [51]. Other studies suggest older individuals may experience less PA due to a more relaxed attitude to life and less COVID-related knowledge among older people [52, 53]. Relationship status is inconsistently related to PA in the general population [43, 46, 54]. Regarding size of household, higher PA was found among individuals living with extended family [43]. With respect to residential size, lower PA levels have been observed in rural areas [49, 50]. Lower income and financial loss are associated with higher PA [22, 46], which could be replicated in an rare disease sample [55]. However, when measuring socioeconomic status (SES) through educational level, the association with PA usually disappears [46, 54, 56]. Also, elevated PA has been linked to previous COVID infections and increased vaccine willingness [54, 57]. To replicate and extend the current state of research, the following hypothesis was derived:

**H2**: Relevant sociodemographic background factors are significantly associated with PA levels among the study sample.

Building on mechanisms described above as well as findings from the general population, indications for additional correlates of PA can be found, which have yet to be tested among patients with rare diseases. These include a consistently observed negative association between HRQOL and PA [58–60]. Also, perceived barriers to healthcare access have been linked to higher PA [57, 61]. Moreover, everyday stressors related to rare diseases such as financial burdens may significantly influence PA [55]. Furthermore, the degree of PA could statistically predict the degree of anxiety and depression disorders in rare disease samples [42, 48]. PA has also been shown to mediate the effects of sociodemographic and other factors on anxiety and depression [53, 62, 63]. A comprehensive understanding of the structure of PA among people living with rare diseases is crucial to improve their situation in the likely event of another pandemic and to prevent negative consequences of maladaptive PA levels as described before. Hence, the following hypotheses were further derived:

**H3**: HRQOL, access to medical resources, and daily burden related to rare diseases are significantly associated with PA levels among the study sample.

**H4**: PA is significantly associated with anxiety and depression levels among the study sample.

**H5**: PA cross-sectionally mediates the links of HRQOL, access to medical resources and daily burden with anxiety and depression levels among the study sample.

## Methods

### Data

Between March 2022 and May 2023, the RESILIENT study (”Retrospective Analysis of the Care Situation and Daily Life of People Living with Rare Diseases During a Pandemic and Derivation of Recommendations for Action”) was conducted at the Department of Medical Psychology at the University Medical Center Hamburg-Eppendorf (UKE), in collaboration with the (German) Alliance of Chronic Rare Diseases (ACHSE) and funded by the Eva Luise and Horst Köhler Foundation. The project used a cross-sectional mixed-methods design; the present study was a secondary analysis focusing exclusively on the quantitative online survey. This survey included standardized as well as newly developed items covering a wide range of relevant areas such as current mental health and worries in connection with COVID-19.

Responses were provided either by affected individuals or, when necessary due to being underage or severely impaired, by parents as caregivers of children with rare diseases. Both questionnaire versions were identical in content, differing only in wording (e.g., “Do you have a disability?” vs. “Does your child have a disability?”). Yet, preliminary regression analysis reveals perspective-based differences in PA, which are controlled for in the structural equation model analysis to account for potential bias. Participants were recruited via fliers distributed digitally through ACHSE’s network of over 130 patient organizations and physically in centers for rare diseases. The only inclusion criterion was a confirmed rare disease diagnosis (self-report). Participation was voluntary, anonymous, and could be withdrawn at any time. The online survey took approximately 30 minutes to complete.

Cases with missing values were excluded from score computation, as no imputation rules were provided in the respective manuals of the standardized scales described below. The proportion of missing data across almost all of the standardized scales and custom-developed items was at 5% or lower. In survey-based research with data usually considered missing at random, such a low level of missingness is generally considered negligible, and complete case analysis is recommended as an appropriate approach. In these cases, imputation methods offer little advantage, as potential bias or loss of precision is minimal and does not materially affect the results [64, 65].

### Measurements

#### Sociodemographic data

Sociodemographic variables include response perspective, gender, age, relationship status, household and residential size, SES, specific rare diagnosis, degree of disability, COVID-19 infection, and COVID vaccination history. All are directly measured through categorical, continuous, or open-format items; except SES, which is calculated using an index based on educational level, income, and occupational status [66].

#### Health-related quality of life

HRQOL is measured using the Short Form Health Survey (SF-8) [67], a brief eight-item version of the SF-36. The SF-8 has shown high reliability (Cronbach’s $\alpha = .85$) and good discriminant validity in large population samples [68]. Higher scores indicate better HRQOL.

#### Access to pandemic-related medical resources

To assess access to medical resources concerning COVID-19, five items are used, covering access to general pandemic-related information, disease-specific information, vaccine-related information, COVID-19 vaccination itself, and medication. The latter had to be excluded in later analyses due to low factor loading (see Sect. 2.3.2). Items regarding information were derived or adapted from the EURORDIS Rare Barometer 2020 [69]; the latter two items were custom-developed. Items vary in format (dichotomous and Likert scales) and are recoded so that higher scores indicate better access to medical resources.

#### Daily burden due to rare disease

**Fig. 1:**
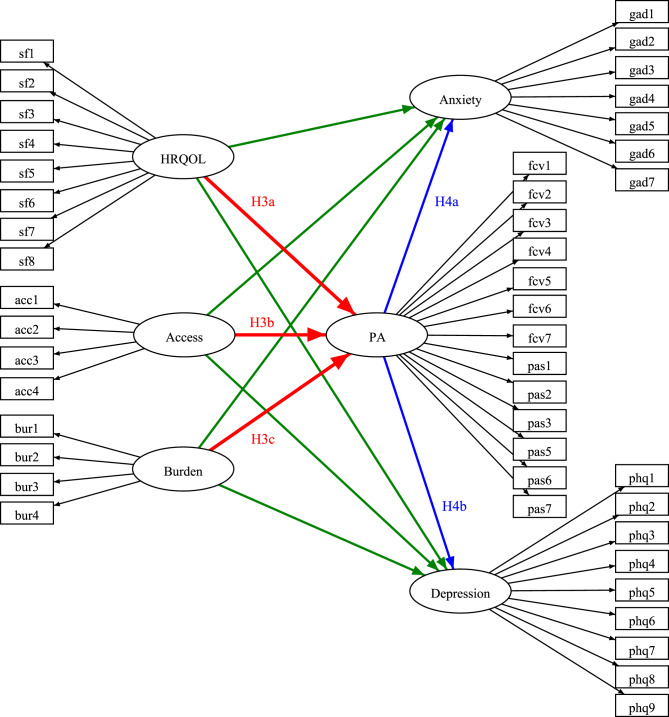
Simplified version of the proposed model, with rectangles representing indicator variables and ellipses representing latent variables. HRQOL: health-related quality of life. PA: Pandemic Anxiety. Not shown: Indirect mediation effects of exogenous variables on anxiety and depression, mediated by PA. To measure daily burden related to rare diseases, it was asked: “To what extent were you affected by the following aspects during the pandemic?”; bur1: “Financial impacts”; bur2: “Little attention paid to the special situation of patients with rare diseases”; bur3: “Having to justify myself because of my situation or being stigmatized”; bur4: “Restrictions in everyday life and lifestyle in general due to the rare disease”. The figure was created using the open-source web application “semdiag” [70]

To assess daily disease-related burden, four custom-developed items are used, measuring financial strain, lack of recognition of disease-specific needs, disease-related stigma, and general daily burden due to the rare disease during the pandemic (for specific item wording, see Fig. 1). All items use a five-point Likert scale, with higher scores indicating greater daily burden.

#### Pandemic anxiety

There are various instruments for measuring the construct of PA. The aim was to measure the latent construct as broadly as possible while at the same time proceeding economically in terms of the online survey. Hence, in this study, PA is measured using the PAS [22] and the Fear of COVID-19 Scale (FCV-19S) [71], as both instruments assess specific aspects. Confirmatory factor analysis of the measurement model (see Sect. 2.3.2) reveals that only one PAS item (”I am concerned that I might infect someone else with COVID-19.”) showed insufficient factor loading (0.35) on the latent factor, which was thus removed from further analyses. Otherwise the FCV-19S and PAS seem to consistently measure a common latent PA factor, thereby covering a wide range of specific aspects.

The PAS, validated in a German representative sample [72], shows acceptable internal consistency (Cronbach’s $\alpha = .73$) and good discriminant validity. The FCV-19S, also validated in a German representative sample [73], demonstrates high reliability (Cronbach’s $\alpha = .81$) and good convergent validity. Short completion times make both scales suitable for survey use. Total scores are calculated by summing item responses. Both scales include seven items rated on a five-point Likert scale, with higher scores indicating greater PA (PAS range: 0 - 28; FCV-19S range: 7 - 35). In conducting complete case analysis for FCV-19S (see Sect. 2.1), we follow previous research [74].

#### Anxiety

Anxiety symptoms are measured using the Generalized Anxiety Disorder Scale (GAD-7) [75], a brief seven-item screening instrument assessing general anxiety, tension, and uncontrollable worry, among other things. The German version [76] shows high reliability (Cronbach’s $\alpha = .89$) and good construct validity. Total score is calculated by summing item responses. All items are rated on a four-point Likert scale, with higher scores indicating greater anxiety. Clinical cutoffs (0 - 4: no anxiety; 5 - 9: mild; 10 - 14: moderate; 15 - 21: severe) have been validated for the German version [76].

#### Depression

Depressive symptoms are measured using the depression module of the Patient Health Questionnaire (PHQ-9) [77], a brief nine-item screening instrument assessing loss of interest, motivation, and suicidal thoughts, among other things. The German version [78] shows high reliability (Cronbach’s $\alpha = .87$) and good construct validity. Total score is calculated by summing item responses. All items are rated on a four-point Likert scale, with higher scores indicating greater depression. Clinical cutoffs (0 - 4: no depression; 5 - 9: mild; 10 - 14: moderate; 15 - 21: severe) have been validated for the German version [78].

### Analyses

#### Group comparisons and regression analyses

The assumption that PA is significantly higher among individuals with rare diseases than in the general population (H1) is tested separately for the PAS and FCV-19S. For each scale, mean sum scores are compared with German population normative scale values [72, 73] from earlier pandemic stages using two Welch t-tests. To adjust for multiple testing, the significance level is Bonferroni-corrected to $\alpha\prime = \alpha/2 = .025$.

To identify relevant sociodemographic background factors associated with PA levels (H2), which should be used as controls in subsequent analyses, two linear regression models are calculated for each sociodemographic factor. This allows for a stepwise descriptive evaluation of mean PA levels as a function of sociodemographic variables (see Table 2). To guard against Type II error (i. e., to not overlook relevant control variables), the significance level is maintained at $\alpha = .05$ despite multiple tests.

The categorical variables gender, age, relationship status, residential size, and specific rare diagnosis are dichotomized prior to regression analyses to reduce complexity and increase ease of interpretation. A power analysis shows that this does not relevantly reduce statistical power of the structural equation model (see Sect. 2.3.2). Dichotomization was carried out based on theoretical and methodological considerations. Regarding gender, all individuals with a diverse gender identity are excluded from subsequent analyses due to very small group size [79]. Regarding age, the sample is divided into individuals under and over 50 years, as the risk for severe COVID-19 courses increases significantly beyond the age of 50 [80]. Regarding specific diagnosis, dichotomization is based on whether the reported rare disease can be explicitly classified as a risk condition for severe COVID-19 or not [29, 30]. However, to prevent information loss due to dichotomization, distributions of gender, age and specific rare diagnoses are reported in full (i.e., as multi-level variables) in Sect. 3.1.

#### Structural equation model

Hypotheses 3 to 5 are jointly tested using a structural equation model. The analysis only includes complete cases without missing values on model-relevant variables ($n=340$; see Sect. 2.1) [81]. An a priori power analysis [82] indicates that, at a significance level of $\alpha = .05$ and with 1118 model degrees of freedom (as specified in the initial model; see below), a minimum of $n = 277$ observations is required to detect a Root Mean Square Error of Approximation (RMSEA) $\ge .02$ with approximately 80% power. Multivariate normality is not met according to Henze-Zirkler test [83]. Thus, all subsequent analyses are conducted using the maximum likelihood mean-adjusted (MLM) estimation method, which provides robust fit indices and Satorra-Bentler-scaled chi-square statistics for non-normally distributed data [84].

Six measurement models specifying the latent constructs (HRQOL, access to medical resources, daily burden related to the rare disease, PA, anxiety, depression) are constructed (see Sect. 2.2). In a first step, confirmatory factor analysis is conducted to assess factor loadings for each measurement model, and all items with loadings $\le .40$ are removed. In a second step, modification indices (MI) are used for each measurement model to improve model fit by specifying additional residual covariances between two indicator variables. The specifications must be theoretically plausible and contribute to a substantial improvement in model fit (MI $\ge 4$) [85, 86]. Model modifications result in excellent robust fit indices for all measurement models [87].

In the final step, an overarching structural model is specified. HRQOL, access to medical resources, and daily disease-related burden are modeled as exogenous latent variables influencing the latent mediator variable PA, which in turn is assumed to affect the endogenous latent variables anxiety and depression. To identify potential mediation effects, both indirect and direct effects of the exogenous on endogenous variables are specified. All sociodemographic background factors significantly associated with PA are included in the model as controls. The model is again optimized by specifying additional residual covariances, if MI $\ge 4$ and if they are theoretically plausible. Figure 1 presents a simplified version of the proposed structural equation model.

## Results

### Descriptive analyses

The online survey was accessed 804 times, with $N = 590$ participants meeting the minimum response criterion. All participants reported to meet the inclusion criterion of at least one confirmed rare diagnosis. 76% ($n = 448$) responded from a self-perspective and 24% ($n = 142$) from an external perspective (e.g., parents of minors or severely impaired persons).

Overall, 68% of participants ($n = 397$) identify as female, 31% ($n = 182$) as male, and 1% ($n = 8$) as diverse; three individuals provided no gender information. 19% of participants ($n = 111$) are between 0 and 17 years old, 9% ($n = 51$) between 18 and 24, 13% ($n = 75$) between 25 and 34, 23% ($n = 134$) between 35 and 49, 28% ($n = 167$) between 50 and 64, and 8% ($n = 45$) are older than 65 years; seven individuals provided no age data. 39% of participants ($n = 230$) declined to specify their diagnosis. Among the 360 who did, 81% report one rare disease, 13% two, and 6% three or more. The most frequently reported primary diagnosis is cystic fibrosis (21%; $n = 75$), followed by rare immunodeficiencies (11%; $n = 39$), sarcoidosis (10%; $n = 37$), and esophageal atresia (8%; $n = 29$). All other reported primary diagnoses affected no more than about 5% of the sample, resulting in a total of 138 unique rare diagnoses among the 360 respondents.

According to self-reports on GAD-7, 34% ($n = 195$) show mild, 15% ($n = 88$) moderate, and 8% ($n = 44$) severe symptoms of anxiety disorder. In contrast, 43% ($n = 242$) report no clinically relevant anxiety symptoms. On PHQ-9, 37% ($n = 207$) report mild, 20% ($n = 112$) moderate, and 16% ($n = 88$) severe symptoms of depression. 28% ($n = 156$) show no clinical signs of depression. The sample’s mean scores are 6.31 ($SD = 4.91$) for GAD-7 and 8.22 ($SD = 5.52$) for PHQ-9, reflecting mild levels of anxiety and depression. See Table 1 for descriptive data on PA and Table 2  for descriptive data on the remaining sociodemographic variables.

**Table 1:** Welch t tests comparing mean PAS and FCV-19S sum scores of the present sample with normative values from German general population

					Comparative statistics			
		$n$	$M$^3^	$SD$	MD [95% CI]	$t$	$df$	$d$
PAS	Present sample	509^1^	11.81	5.69	1.18***$[0.62, \infty]$	3.45	1014.70	0.22
	Norm sample^2^	515	10.63	5.29				
FCV-19S	Present sample	517^1^	15.87	5.87	2.76***$[2.27, \infty]$	9.24	870.30	0.55
	Norm sample^2^	866	13.11	4.45				

*Notes*. All decimal numbers are rounded to two decimal places. PAS: Pandemic Anxiety Scale (range: 0–28). FCV-19S: Fear of COVID-19 Scale (range: 7–35). MD [95% CI]: Mean difference with one-sided 95% confidence interval. $d$: Effect size. Interpretation based on Bonferroni-corrected significance level of $\alpha\prime = .025$

^1^PAS and FCV-19S subsamples after exclusion of all cases with missing values

^2^Sample size, mean, and standard deviation derived from the German PAS norm sample [72] and from the German FCV-19S norm sample [73]

To evaluate precision of mean estimates, corresponding standard errors of the mean were calculated (PAS present sample: 0.25; PAS norm sample: 0.23; FCV-19S present sample: 0.26; FCV-19S norm sample: 0.15). All standard errors of the mean are small relative to the respective SDs, indicating a high precision of the mean estimates

***$$p < .001$$

**Table 2:** Descriptive statistics and identification of relevant sociodemographic background factors using linear regression analyses

		Pandemic Anxiety			
		PAS^1^		FCV-19S^1^	
	$n (\%)$	$M (SD)$	$B (SE)$	$M (SD)$	$B (SE)$
**Gender**^2^					
male	182 (31.4)	10.89 (5.56)	Reference	14.47 (5.76)	Reference
female	397 (68.6)	12.18 (5.68)	1.29* (0.55)	16.47 (5.83)	2.00*** (0.56)
**Age**^3^					
$<$ 50 years	371 (63.6)	11.75 (5.80)	Reference	15.18 (5.63)	Reference
$\ge$ 50 years	212 (36.4)	11.91 (5.55)	0.16 (0.53)	17.01 (6.07)	1.83*** (0.53)
**Relationship status**^4^					
marriage/partnership	420 (71.4)	11.85 (5.59)	Reference	16.03 (5.98)	Reference
single/divorced/widowed	168 (28.6)	11.84 (5.90)	−0.01 (0.56)	15.59 (5.53)	−0.44 (0.57)
**Residential size**^5^					
rural ($<$ 20,000 res.)	241 (41.1)	12.19 (5.31)	Reference	16.26 (5.81)	Reference
urban ($\ge$ 20,000 res.)	346 (58.9)	11.55 (5.92)	−0.64 (0.51)	15.96 (5.88)	−0.66 (0.52)
**Rare diagnosis**^6^					
no risk diagnosis	54 (15.0)	12.93 (5.65)	Reference	15.74 (6.15)	Reference
risk diagnosis	306 (85.0)	11.35 (5.55)	−1.58 (0.89)	15.38 (5.65)	−0.36 (0.90)
**Degree of disability**^7^					
no	154 (26.5)	11.52 (5.33)	Reference	15.17 (4.82)	Reference
yes	427 (73.5)	11.90 (5.83)	0.38 (0.58)	16.09 (6.15)	0.92 (0.59)
**Prior infection**^8^					
no	301 (56.7)	12.08 (5.79)	Reference	16.63 (6.24)	Reference
yes	230 (43.3)	11.51 (5.52)	−0.57 (0.51)	14.86 (5.20)	−1.77*** (0.52)
**Vaccination**^9^					
no	58 (12.4)	10.97 (5.75)	Reference	13.86 (5.56)	Reference
yes	411 (87.6)	11.88 (5.63)	0.91 (0.79)	16.08 (5.80)	2.22** (0.82)

	$M (SD)$	$B (SE)$		$B (SE)$	
**Number of people in household**^10^	2.57 (1.22)	−0.19 (0.21)		−0.48* (0.21)	
**Socioeconomic status**^11,12^	13.03 (4.33)	−0.06 (0.06)		−0.11 (0.06)	

*Notes*. All decimal numbers are rounded to one or two decimal places. Valid percentages are reported excluding missing values; for number of missing cases, see notes 2–11

^1^Dependent variables of regression analyses: mean sum scores of Pandemic Anxiety Scale (range: 0–28) and Fear of COVID-19 Scale (range: 7–35) ^2^$n = 11$^3^$n = 7$^4^$n = 2$^5^$n = 3$^6^$n = 230$^7^$n = 9$^8^$n = 59$^9^$n = 121$^10^$n = 12$^11^$n = 15$^12^3 - 8: lower class; 0 - 14: middle class; 15 - 21: upper class [66]

*$$p < .05$$; **$$p < .01$$; ***$$p < .001$$

### Levels of pandemic anxiety

As shown in Table 1, both PAS and FCV-19S mean sum scores are significantly elevated compared to normative data from representative samples [72, 73]. Effect sizes are small for PAS ($d = 0.22$) and moderate for FCV-19S ($d = 0.55$), supporting hypothesis 1.

### Sociodemographic background factors associated with pandemic anxiety

Higher PA is observed among females, individuals of age 50 and older, those never infected, and those vaccinated; PA decreases with more people in the household (see Table 2). Except for gender, these effects appear only for FCV-19S, not PAS. Relationship status, residential size, SES, specific rare diagnosis, and degree of disability are not meaningfully related to PA. Thus, hypothesis 2 is supported in regard to gender, age, number of people in household, and infection and vaccination history, and must be rejected in regard to the remaining sociodemographic variables.

### Structural equation model

**Fig. 2:**
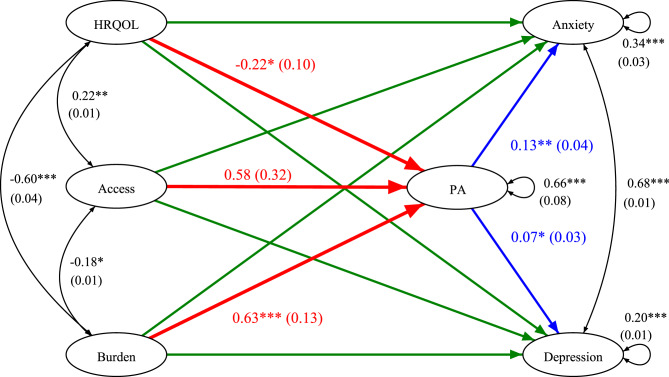
Simplified version of the proposed model, showing unstandardized path coefficients (and standard errors) of specified effects. All numbers are rounded to two decimal places HRQOL: Health-related quality of life. PA: Pandemic Anxiety. Not shown: Indirect mediation effects of exogenous variables on anxiety and depression, mediated by PA. Unstandardized path coefficients (and standard errors) of green effects from top to bottom: −0.64*** (0.07); 0.19 (0.17); 0.03 (0.06); −0.65*** (0.06); −0.15 (0.13); −0.02 (0.04). All effects involving PA are controlled for gender, age, household size, previous COVID-19 infection and vaccination, and response perspective. The figure was created using the open-source web application “semdiag” [70]

The structural equation model is based on $n = 340$ complete cases with no missing data on any model variables. Satorra-Bentler scaled chi-square statistic ($\chi^2 = 1721.61, p < .001$) indicates significant misfit for the modified model; however, this likely reflects the test’s sensitivity in large samples rather than poor model fit. As recommended for larger samples, additional fit indices are used to assess overall model quality [88, 89]. According to standard conventions for fit indices [87], the model fits the data well (CFI = 0.923; TLI = 0.911; RMSEA [90% CI] = 0.043 [0.039, 0.047]; SRMR = 0.077). The only unmet criterion is CFI $\ge .95$. This may be due to the fact that incremental fit indices like CFI penalize model complexity and tend to decline as the number of estimated parameters increases [89]. In summary, the model demonstrates good fit to the data, making it suitable for testing hypotheses 3 to 5. Figure 2 presents the simplified model including all relevant parameters. For reasons of clarity, indicator variables, their residual variances and covariances, as well as factor loadings, are not displayed.

The latent standardized residual variances indicate that 66% of the variance in PA remains unexplained by the model ($R^2~=~.34$), suggesting that additional factors beyond HRQOL, access to medical resources, and daily burden due to rare diseases contribute to PA in the sample. Furthermore, the model explains 66% of variance in anxiety scores ($R^2~=~.66$) and 80% of variance in depression scores ($R^2~=~.80$). The latent standardized covariances show significant associations between latent factors for which no dependencies were specified in the model. Better access is linked to higher HRQOL and lower burden, and lower burden is also associated with higher HRQOL. Anxiety and depression scores are strongly correlated.

The model controls for gender, age, household size, COVID-19 infection and vaccination history, as well as response perspective. The other sociodemographic PA-related factors are excluded from the model since no significant effects on PA (neither PAS nor FCV-19S) could be found (see Sect. 3.3). Significant associations are found between HRQOL and PA ($B$ = −0.22, $\beta$ = −0.17, $z$ = −2.40, $p$ = 0.016), daily disease-related burden and PA ($B$ = 0.63, $\beta$ = 0.40, $z$ = 4.79, $p < .001$), as well as between PA and both anxiety ($B$ = 0.13, $\beta$ = 0.18, $z$ = 3.18, $p$ = 0.001) and depression ($B$ = 0.07, $\beta$ = 0.11, $z$ = 2.32, $p$ = 0.020). Access to medical resources is not significantly associated with PA in the current sample ($z$ = 1.81, $p$ = 0.070). Thus, there is partial support for hypothesis 3 and full support for hypothesis 4.

Daily disease-related burden shows significant indirect effects (not shown in Fig. 2), via PA, on both anxiety ($B$ = 0.08, $\beta$ = 0.07, $z$ = 2.62, $p$ = 0.009) and depression ($B$ = 0.04, $\beta$ = 0.04, $z$ = 2.15, $p$ = 0.032). Since the corresponding direct effects of burden on anxiety ($z$ = 0.53, $p$ = 0.598) and depression ($z$ = −0.56, $p$ = 0.578) are not significant, the impact of daily disease-related burden on anxiety and depressive symptoms is clearly mediated by PA, partially supporting hypothesis 5. The remaining four indirect effects of HRQOL and access on anxiety and depression, via PA, are not statistically significant (for all: $|z| \le 1.94$, $p \ge .053$). Thus, parts of hypothesis 5 must be rejected, as no significant mediation effects of HRQOL or access on anxiety and depression via PA were found in the current sample.

## Discussion

People living with rare diseases experience higher levels of PA than the German general population. Women, individuals of age 50 and older, and those living alone report particularly high PA. A prior COVID-19 infection is associated with lower PA, vaccination is linked to higher PA. Decreased HRQOL and increased daily disease-related burden are associated with elevated PA. Increased PA is also associated with higher anxiety and depression scores on clinical screening measures. Moreover, PA cross-sectionally mediates the relationship between daily disease-related burden and both anxiety and depression.

### Integration of results

The clear surplus of women among the participants contradicts the usually balanced gender ratio among patients with rare diseases in large population-based studies [33]. This can be explained by the fact that women are more likely than men to participate in health prevention initiatives [90] and that at least 24% of respondents are caregivers for children with rare diseases, roles predominantly filled by women [91]. Compared to a population-based study [33], adults over 65 are underrepresented, likely due to lower engagement with online surveys. Cystic fibrosis emerged as the most common diagnosis, consistent with being the most common rare disease in Europe [26]. The identification of 138 unique rare diagnoses among 590 participants reflects the rarity paradox (many rare diseases, but few cases per condition) [23]. Although not a psychiatric sample, the mild average GAD-7 and PHQ-9 scores align with known elevated mental health risks in rare disease populations [25]. 23 and 36% of participants report moderate to severe anxiety or depression, allowing for substantial variance in symptom severity to be partially explained by PA.

As proposed in hypothesis 1, PA scores on both scales are significantly higher than German population normative values, replicating previous findings [42] in a larger rare disease sample. While no validated cutoffs exist for the German versions of PAS or FCV-19S, international studies suggest FCV-19S thresholds of 16.50 and 17.50 for clinically relevant PA [92, 93]. The mean FCV-19S score in this sample (15.87) falls just below those thresholds, though cultural differences limit direct comparisons. The mean FCV-19S score of 15.87 is also lower than in prior studies on the general population (18.57) [44] and another sample with rare diseases (20.20) [51]. This is likely due to the later timing of data collection in the current study (from March 2022), when PA had begun to decline corresponding to successive containment of the pandemic [94, 95].

As proposed in hypothesis 2, among all patients with rare diseases, women and individuals of age 50 and older experience particularly high PA. This is in line with current research (see Sect. 1). These findings imply that special attention should be given to women and older individuals with rare diseases in the event of future pandemics. PA decreases with the number of people per household. While higher PA is found in people living with extended instead of core family [43], households in the current sample averagely comprise two to three people (with an inter-quartile range of 1), meaning the variable primarily differentiates between people living alone and with core family. That said, the result is in line with findings of elevated mental stress in people living alone during the pandemic [96]. It implicates that in future pandemics, targeted measures against household loneliness, such as volunteer-based domestic support, should be considered.

The finding of reduced PA among people who had previously been infected with SARS-Cov-2 is unprecedented among samples with rare diseases. Considering the high risk of severe COVID courses among these patients, it seems counterintuitive. It might be explained by mechanisms of habituation [97], which, however, cannot be confirmed on the basis of the present data. The significant association between vaccination and higher PA aligns with prior findings of moderate PA increasing preventive health-related behaviors within the framework of the Expanded Health Belief Model [57]. In another study, functional fear could predict public health compliance (e.g., hand hygiene) during the COVID-19 pandemic [98]. Attempting to synthesize the present findings on infection and vaccination history, it could be concluded that anxiety-prone individuals should be more likely to seek vaccination as a coping strategy. In contrast, those who have already experienced infection might exhibit reduced anxiety due to habituation, resulting in lower proactive health engagement.

The remaining sociodemographic background factors are not significantly associated with PA in the present sample. PA appears to be more strongly influenced by the physical living situation than by relationship status, residential size and SES. The present sample primarily represents middle to upper socioeconomic groups rather than the lower class (see Table 2), which may explain why SES does not significantly influence PA levels in the present sample.

As proposed in hypothesis 3, lower HRQOL and higher daily disease-related burden are both associated with elevated PA across a broad range of diseases, even when controlling for relevant sociodemographic factors. This aligns with findings of generally reduced HRQOL [24, 27] and increased daily burden [27, 28, 41] among patients with rare diseases, both before and during the pandemic, and extends those findings by clearly identifying these associated factors of PA. In advance of a future pandemic, reducing PA among people living with rare diseases may be achieved both by improving overall HRQOL, i. e. through better relieving chronic pain and increasing energy levels, as well as by challenging daily stressors, i. e. through raising public awareness for the unique challenges faced by patients with rare diseases and actively combating disease-related stigma.

Contrary to hypothesis 3 and prior findings in the general population [57, 61], access to pandemic-related medical resources is not meaningfully associated with PA in the present sample. This may be largely due to strong sample selection bias, since participants were solely recruited via patient organizations and specialized care centers. Descriptive analyses of items used to measure access (see Sect. 2.2.2) confirm that the sample predominantly consists of individuals well integrated into the healthcare system, with high reported access to information and vaccination. Additionally, access to COVID-19 vaccination is exceptional in that it did not worsen during the pandemic due to rare diseases patients’ prioritized eligibility [?]. As half of the access indicators focus on vaccination, this likely further reduced predictive power.

As proposed in hypothesis 4, PA is significantly associated with anxiety and depression scores in the present sample, even when controlling for relevant sociodemographic factors. This supports earlier research in the general population [99] and extends limited findings in groups with rare diseases [34, 42, 48]. The findings highlight the importance of investigating PA as a serious mental health risk. While moderate PA may be adaptive in accordance to the Expanded Health Belief Model [57], its significant link to clinical anxiety and depression suggests a threshold above which PA becomes maladaptive for mental health [44, 46]. Since pandemic-related mental disorders can outlast pandemic time-frames [10], PA should not be dismissed as a temporary pandemic state, but recognized as a potential contributor to lasting psychological distress.

As proposed in hypothesis 5, PA cross-sectionally mediates the link between daily disease-related burden and both anxiety and depression scores. This extends prior findings from the general population [53, 62, 63]. The findings further highlight the need for challenging daily stressors to mitigate PA and, subsequently, anxiety and depression. Contrary to hypothesis 5, PA does not mediate the relationships between HRQOL and anxiety or depression in the present sample. However, significant direct effects were found, which aligns with previous findings in the general population [100, 101]. These direct effects may be so strong that the mediating role of PA is not relevant in this context. Further, neither direct nor indirect effects of access to medical resources on mental health outcomes are observed. This indicates a flawed theoretical assumption [102], likely due to the limited variance in access-related variables and the high degree of healthcare integration within the sample.

### Strengths and limitations

The present study integrates both clinical measures and validated PA scales, making it the first to systematically examine the extent and structure of PA across a large and diverse sample of people living with rare diseases. In doing so, it contributes to the ongoing discussion around a biopsychosocial model of PA, which has so far been limited by methodological constraints when proposed for the general population [46]. By synthesizing prior findings and applying them to the present sample, the present study proposes and empirically supports a PA model incorporating somatic, psychological, and social factors. While findings are generalizable due to sample size and complexity of the structural equation model, they should be interpreted with caution given certain limitations.

A key limitation lies in selection bias during sample recruitment. As participants were only recruited via patient organizations, specialized clinics, or centers for rare diseases, the sample over-represents individuals well integrated into healthcare. This may explain why access to healthcare was not identified as a relevant correlate of PA in this study. Those with undiagnosed conditions, limited access to care, or heightened social isolation during the pandemic are most likely under-represented. Another limitation is that the diagnoses could not be objectively verified due to the online format, a common issue in research in these samples. Given the variety of rare diseases, not all diagnoses are represented in the present sample. Additionally, all data are based on self-report measures, which increases susceptibility to biases such as socially desirable response patterns [103]. Also, GAD-7 and PHQ-9 are screening tools merely indicating the existence of mental disorders, and they are not equivalent to clinical psychiatric diagnoses [104].

A further limitation concerns the measurement of PA by combining items from the PAS and FCV-19S, two scales with different latent factor structures. While this raises questions about construct validity, both scales equally capture the central PA symptom of fear of personal health threats [21, 22, 105], and almost all items loaded significantly on a common latent factor when specifying the structural equation model (see Sect. 2.2.5). Including both scales also improves reliability [106]. Additionally, PA data were collected later than the scale normative values used for comparison, which may introduce bias due to differing contextual factors. However, PA remained significantly higher in our sample despite progressing containment of the pandemic.

The most important limitation is that structural equation modeling based on cross-sectional data cannot establish causality. Causal claims require both temporal ordering and control of confounding variables [85]. It could be argued that PA is defined as pandemic-specific and thus temporally bounded, whereas factors like HRQOL, access, and daily burden were already relevant before the pandemic, and clinical anxiety or depression often persist beyond it. These theoretical considerations support the proposed directionality despite the cross-sectional design, and the findings offer some empirical support. Nonetheless, all variables were measured simultaneously, limiting causal inference. Reverse causality remains plausible for nearly all of the significant associations between PA and other variables (see, for example, the finding on PA predicting health compliance in Sect. 4.1). Also, a well-fitting model does not rule out alternative explanations or reversed pathways [107]. Therefore, findings should primarily be interpreted as correlational. Furthermore, it should be noted that statistically significant results do not necessarily indicate clinically significant changes. To address limitations, future research directions are outlined below.

### Implications for future research

The most effective way to strengthen causal conclusions is through longitudinal research. This would allow for clearer insights into which factors causally influence PA in people living with rare diseases, and how PA in turn affects mental health outcomes. Such studies do not necessarily need to be embedded within an ongoing pandemic context, as they could retrospectively assess pandemic-related experiences and track mental health over time to evaluate the long-term impact of PA on disorders like anxiety. Compared to data collected during a pandemic, retrospective assessments are less valid. Thus, patient-reported variables beyond HRQOL should ideally be included in rare disease registers. Repeated measurements based on those data would yield pre-, peri-, and post-pandemic data points for different groups. Comparing a pre–peri difference with a pre–pre difference between two rare disease groups would allow for causal conclusions about true pandemic effects, given that outcomes are measured consistently over time [108].

Hereby including a control or comparison group (e.g., individuals with common chronic diseases or other complex diseases) would clarify whether patients with rare diseases are more severely affected than others. Furthermore, differences within the group of patients with rare diseases could be investigated by comparing specific rare disease groups (e.g., immunodeficiency, neurological disorders). This would enable targeted policy recommendations, identifying which causal factors should be addressed preemptively to prevent elevated PA and its negative short- and long-term consequences in future pandemics.

In light of the findings on infection and vaccination history, which in part seem counter-intuitive (see Sect. 4.1), future studies should include measures on whether prior infections have indeed induced anxiety to a relevant degree, in order to potentially replicate and explain the association between a prior COVID infection and decreased PA. Also, developing and using standardized PA scales with valid cut-off scores would help differentiate between functional and maladaptive PA levels, thereby adding explanatory depth to the positive link between a prior vaccination and PA.

Recruitment strategies of future studies need to be extended beyond contexts specific to rare diseases (such as specialized clinics and patient organizations), to reach those less integrated into the healthcare system. Future sampling should also consider stratification by gender and SES to improve representativeness, and use oversampling strategies to include individuals beyond the cis-binary gender system.

Several additional correlates of PA, previously found relevant in the general population, warrant further investigation in the context of rare diseases. Social support has shown a negative association with PA in some studies [54], though not all confirm this [46, 51]. Perceived COVID-19 threat is a consistent predictor of both PA and related psychopathology, with PA acting as a mediator [62]. Health literacy may buffer PA levels, as supported by several studies [54, 109], though not among students [110]. Excessive use of social media as a primary COVID-19 information source has also been linked to increased PA and depression [63, 111]. The present model should be expanded to include all previously mentioned factors. In doing so, future longitudinal research could help develop a more elaborate and causal biopsychosocial model of PA and its consequences during a pandemic and beyond, which is especially relevant given the high likelihood of future pandemics and the increased vulnerability of people living with rare diseases.

## Conclusion

Since the COVID-19 pandemic is currently relatively well contained, the main value of the present results is of preventive nature. Based on robust findings, it clearly identifies specific factors that health policy should target to effectively improve the situation of the vulnerable subpopulation of people living with rare diseases in the likely event of a future pandemic. The present study takes the first step toward developing a biopsychosocial model of PA for patients with rare diseases. It shows that among this group, women, individuals of age 50 and older, and those living alone are particularly affected by PA during the COVID-19 pandemic. These groups should receive special attention in future pandemics. While PA is linked to greater willingness to vaccinate, it is also associated with increased anxiety and depression that may persist beyond the pandemic. PA should therefore not be dismissed as a temporary pandemic reaction. The analysis also finds that higher HRQOL and lower daily disease-related burden are associated with lower PA. Thus, improving physical health, managing pain, raising public awareness for rare diseases, and reducing disease-related stigma should be prioritized in preparation of future health crises to mitigate maladaptive PA at those times.

## Data Availability

The underlying data are not publicly available due to privacy reasons but are available from the corresponding author on reasonable request and after consultation with the local data protection manager.

## Declarations

### Generative AI and AI-assisted

During the preparation of this work, the author HS used ChatGPT-4o and DeepL in order to improve readability and language of the work. After using this tool, the authors reviewed and edited the content as needed and take full responsibility for the content of the publication.
